# Visual4DTracker: a tool to interact with 3D + t image stacks

**DOI:** 10.1186/s12859-020-03820-y

**Published:** 2021-02-08

**Authors:** Ermanno Cordelli, Paolo Soda, Giulio Iannello

**Affiliations:** Unit of Computer Systems and Bioinformatics, Department of Engineering, Università Campus Bio-Medico di Roma, Rome, Italy

**Keywords:** Tracking, Image proofreading, Blobs, MATLAB package

## Abstract

**Background:**

Biological phenomena usually evolves over time and recent advances in high-throughput microscopy have made possible to collect multiple 3*D* images over time, generating $3D+t$ (or 4*D*) datasets. To extract useful information there is the need to extract spatial and temporal data on the particles that are in the images, but particle tracking and feature extraction need some kind of assistance.

**Results:**

This manuscript introduces our new freely downloadable toolbox, the *Visual4DTracker*. It is a MATLAB package implementing several useful functionalities to navigate, analyse and proof-read the track of each particle detected in any $3D+t$ stack. Furthermore, it allows users to proof-read and to evaluate the traces with respect to a given gold standard. The Visual4DTracker toolbox permits the users to visualize and save all the generated results through a user-friendly graphical user interface. This tool has been successfully used in three applicative examples. The first processes synthetic data to show all the software functionalities. The second shows how to process a 4*D* image stack showing the time-lapse growth of Drosophila cells in an embryo. The third example presents the quantitative analysis of insulin granules in living beta-cells, showing that such particles have two main dynamics that coexist inside the cells.

**Conclusions:**

Visual4DTracker is a software package for MATLAB to visualize, handle and manually track $3D+t$ stacks of microscopy images containing objects such cells, granules, etc.. With its unique set of functions, it remarkably permits the user to analyze and proof-read 4*D* data in a friendly 3*D* fashion. The tool is freely available at https://drive.google.com/drive/folders/19AEn0TqP-2B8Z10kOavEAopTUxsKUV73?usp=sharing

## Background

Microscopy images are widely used to study biological phenomena since they can provide a variety of useful information about morphology, structure, functional issues, and dynamics. In particular, dynamic investigations make use of time-lapse microscopy, where microscope image sequences are recorded and then observed at a greater speed to give an accelerated view of the process. This technique permits the study of space and time evolution of sets of particles, such as cells, granules or vesicles (hereinafter also referred to as blobs). Particle tracking, which consists in blobs’ localization and trajectories’ reconstruction, is a basic step useful also to extract quantitative features from image series [1–7].

Although biological phenomena have in general a 3*D* nature, time-lapse experiments traditionally collect a series of 2*D* images over time, referred to as $2D+t$ datasets. However this approach may lose relevant 3*D* information and, to overcome this limitation, recent advances in high-throughput microscopy have made possible to image multiple *z*-positions of the sample in a time that is at least an order of magnitude smaller than the time scale of the biological process. This makes possible to collect 3*D* image stacks of the sample at different times, generating $3D+ t$ datasets (also referred to as 4*D* in the following). These datasets are typically fairly large and their size tends to further grow as long as acquisition technologies improve. Moreover, in many cases in the field of view of the acquisition system there is a large number of particles, sometimes of more types, whose spatial and temporal behavior should be extracted and properly characterized to derive the information of interest. Particle tracking and feature extraction in these cases necessarily require some kind of assistance. For these reasons several commercial and open source software tools have been developed to handle $2D+t$ and $3D + t$ datasets, and to assist biologists in the extraction of quantitative information. In this respect, this manuscript presents *Visual4DTracker*, a software package for MATLAB handling $3D + t$ stacks and offering the possibility to proofread the data in 3*D*. It overcomes the main limitations of open source tools currently available, as described in the following sections.

### Computational methods

We will focus here only on $3D+t$ particle tracking, as $3D+t$ datasets may carry more information on the biological processes under investigation than $2D+t$ data volumes. To the best of our knowledge, DiaTrack [8], ilastik [9] and TrackMate [10] are the most popular open source tools performing automatic tracking of multiple particles, whose main properties are summarized in Table 1. Furthermore, DiaTrack and TrackMate permit the computation of a predefined set of quantitative features from blob traces (e.g. velocity, acceleration, etc).

**Table 1:** Summary of the main properties of the open source softwares for 4*D* tracking

Software	ilastik [9]	DiaTrack [8]	TrackMate [10]	Visual4DTracker (proposed)
Manual tracking	YES: after segmentation, clicking frame by frame the moving object	NO	NO	YES
Automatic tracking	YES: it uses the Least Square, Distance association method	YES: after segmentation, it uses the Least Square Distance association method	YES: after segmentation, it uses the Least Square Distance association method	NO
Single particle tracking	YES	NO	NO	YES
Multiple particle simultaneous tracking	YES	YES	YES	NO
Particle occlusion handling	YES: it is possible to manually define split events of single objects	NO	YES: it automatically searches for multiple objects split events	NO
Pre-processing	YES (several filters)	YES (only Gaussian Filter, luminosity threshold)	NO	YES (several filters, user defined functions)
Proof-reading	NO	NO	YES: it is only possible to load the traces and to modify the tracking parameters	YES
Tracking processing	NO	YES: it is possible to compute kinematic parameters from the traces	NO	NO
3D tracking visualization	NO: only 2D multiple orthogonal layers representation	NO: only 2D multiple orthogonal layers representation	NO: 2D multiple layers representation surfable in quote and frames	YES
3D data reconstruction	YES	YES	YES	YES
Manual tracking in 3D visualization	NO	NO	NO	YES
User developped pre/post processing functions	NO	NO	NO: not directly, however the software is a Fiji plug-in	YES: it is possible to upload custom functions in MATLAB
Output	File in .hdf5 format (or others)	File in .txt format trajectories and data and .eps format for the images	File in .xml format	File in .xml format

On the one side, these tools provide the possibility to rotate, zoom and surf the 3*D* volume at a given *t*; on the other side, fixed the view of the volume in terms of zoom, angle of view, rotation and pan, the users can surf its time evolution. However, they lose the ability to show the 3*D* view when the data has to be annotated: in this case, the user is limited to work with the orthographic projections on the frontal, lateral and horizontal vistas. The use of an own data structure for storing the tracking results as well as any other computed quantitative data is a common limitation of such tools. Straightforwardly, this prevents data sharing between different softwares, platforms and, eventually, researchers.

In many practical applications, once a tool has automatically provided the particle traces, researchers and practitioners may need to proofread these data, as the results can be far to be fully satisfactory. In this respect, only TrackMate offers this functionality, but the user has to interact only with 2*D* views of the $3D+t$ data. Moreover, there is no possibility to proofread traces computed by other tools. In other cases the users may need to manually track a particle or a limited set of particles: to this aim, a researcher can also try to use other open source tools, such as Fiji or Vaa3D [11], but still there are some limitations. For instance, in Fiji the interaction is again only with 2*D* views of the $3D+t$ data, while in Vaa3D there is no chance to perform a frame-to-frame blobs’ tracking although the software allows an easy $3D + t$ interaction with the data. In fact, one of the purposes of Vaa3D project is to easily visualize and analyse 3D/4D/5D acquisitions, providing the user with specific tools to interact with the images. However, all elaborations are focused on single frames, thus making the software poorly adaptable for a tracking task.

The software package we are presenting in this manuscript can handle $3D+t$ stacks, and it can be used as a tool to proofread 3*D* data.

In a nutshell, the software shows the stack sequences into a 3*D* space, and a graphical user interface (GUI) allows users to navigate and to interact with the data in space and time. The users can therefore manually trace blobs, proofread an existing trace, view all the traces directly in 3*D*, and store the results in an *xml* file formatted^1^ to facilitate data sharing. Moreover, to facilitate the integration with other tracking softwares, we provided Visual4DTracker the possibility to load DiaTrack’s and ilastik’s tracking output file formats i.e. *.mat* and *.csv* extensions respectively,^2^ in a completely transparent manner to the user. Although the proposed tool does not allow an automatic tracking process, it is still worthy as it can bring particular benefits to the entire manual tracking procedure, by giving the opportunity to the user to enjoy the natural time evolution of each analysed volume using the most intuitive and easy to follow 3*D* representation of the data. Finally, the possibility to proofread external tracking results and to further evaluate them against a ground truth brings a considerable relief for the user’s job.

## Implementation

**Fig. 1:**
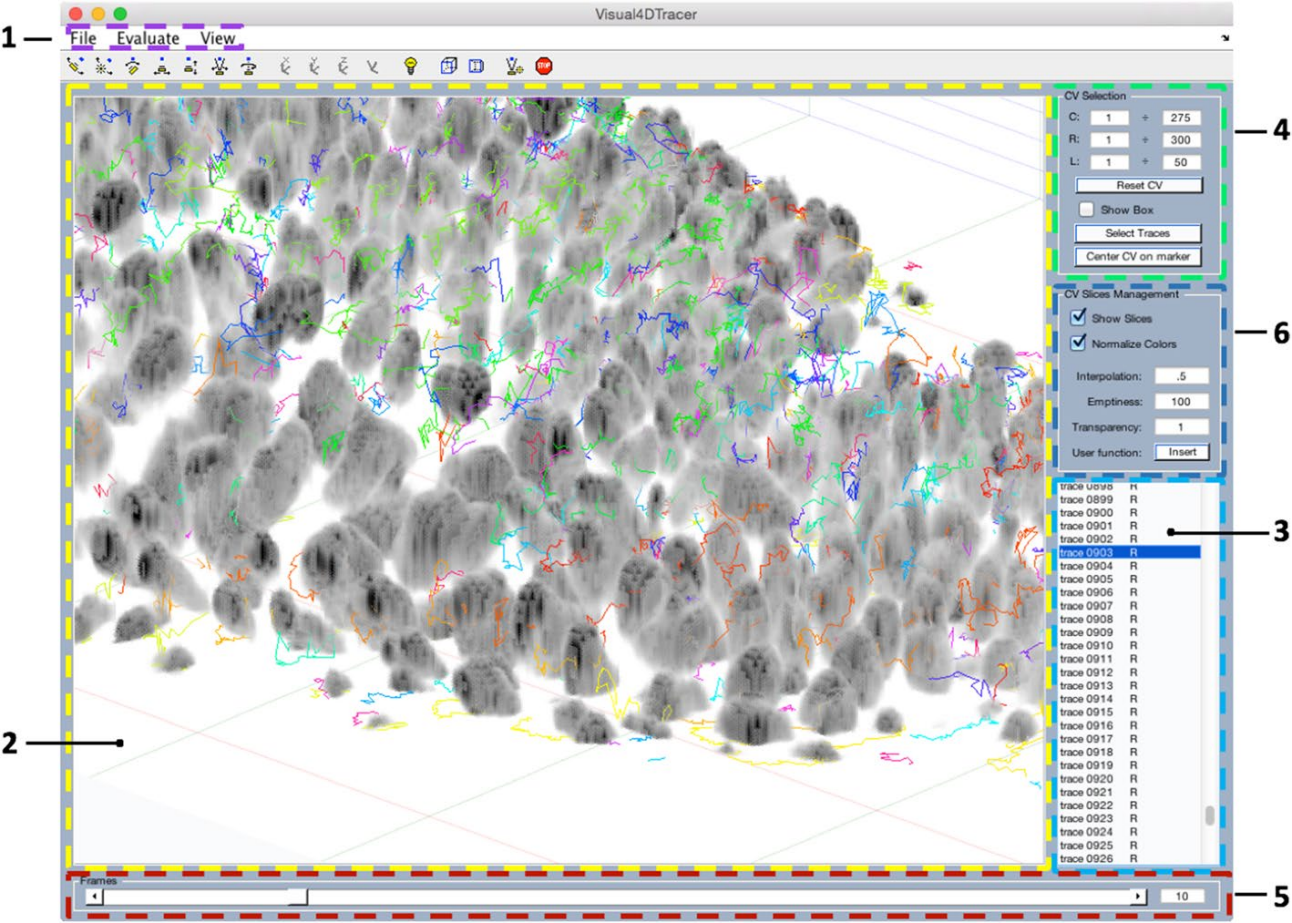
Main GUI’s appearance: the numbers labels the functional panels: 1—list menus; 2—main panel; 3—trace panel; 4—CV selection panel; 5—frame panel; 6—CV slice management panel. This example shows the 3D representation of Drosophila cells growing inside a larva, where each trace over the time is represented with a different color. The Drosophila dataset can be downloaded at http://ilastik.org/download.html

For an easier comprehension Fig. 1 shows the main GUI’s appearance where the functional panels are labeled with numbers.

Visual4DTracker is an open source software developed as a MATLAB package. It allows to handle, to visualize and to surf 4*D* stacks of images, containing objects moving in a 3*D* space and acquired over time at a microscope. Figure 1 shows the appearance of the tool GUI where the functional panels are labeled with numbers. Using the menu marked by (1), the user can mark the objects’ positions frame by frame and to save their traces in a file that can be exported. Moreover, there is the possibility to load a file containing a set of traces and to proceed further with data annotation. This functionality yields the possibility to proofread traces already marked by other users or provided by a software for automatic tracing, thus being able to compare two different traces belonging to the same experiment. The top menu allows also the user to set several markers and traces graphical properties. For example, the user has the possibility to switch from dimensionless markers, useful when dealing with numerous small objects e.g. cells’ granules, to spherical volumetric markers, that can help to easily identify blobs with a larger size, e.g. cells.

Visual4DTracker consists in a main window (MW), marked by (2) in Fig. 1, where the 3*D* volume collected at a given time stamp is shown. The different time stamps can be accessed using a slider located at the bottom of the GUI, which is denoted as (5) in Fig. 1. The volume can be rotated, zoomed and panned using the mouse, the keyboard or a set of specific buttons in the toolbar. Other graphical objects at the bottom and at the left of the MW allow the user to interact with the 4*D* data. On the right side there are three panels. The first ((4) in Fig. 1) permits users to visualize a parallelepiped shaped ROI in the MW, helping user focus the attention to a certain volume, and freeing up the memory to improve the user experience. The second panel ((6) in Fig. 1) offers some basic image processing functionalities to reduce noise, to improve the contrast in the volume, and to resample the stack. The third panel ((3) in Fig. 1) lists either the traced blob traces or those loaded from an external file. The user can select which trace has to be shown, and the visualization engine shows the trace as a whole together with a subset of its markers (eventually all).

**Fig. 2:**
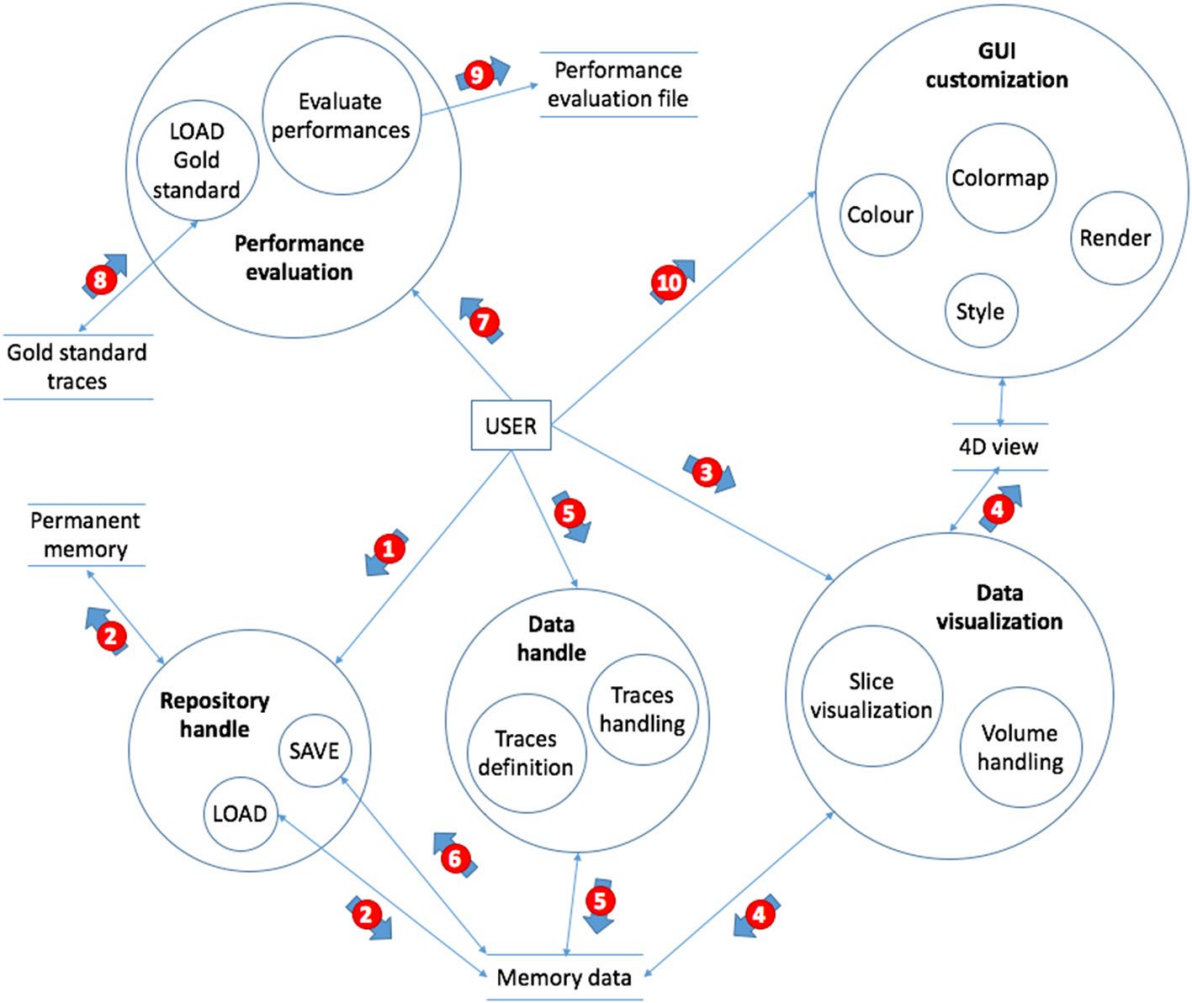
Tool’s data flow diagram: the circles represent each process of the GUI linked each other with slim arrows, the coupled horizontal lines symbolize data repositories and the wide arrows with number show the typical actions and data flow

Next subsections describe the main characteristics of the software, while the supplementary material provides a detailed description. Additionally, Fig. 2 offers a graphical representation of Visual4DTracker structure and interfaces via a data flow diagram, where the data transformation circles (i.e. the circles in light blue) represent each functionality of the GUI. The primary entity is the user, who can interact with the internal functionalities of the system: indeed he/she can handle the image repository, handle the data annotation process, manage data visualization, customize the GUI, and evaluate the tracking performance between annotated traces and gold standard ones. In the data flow diagram such functionalities are connected each other by slim arrows, representing the basic data that must flow between these top-level functions. Furthermore, horizontal parallel lines represent data stores. While this graphical representation of hierarchical breakdown and interfaces provides a good view of the component model, it offers a poor view of the transactions from input to output (or to complete some system actions); for this reason in Fig. 2 we also add the light blue thick numbered arrows that illustrate the typical flow of actions and data due to the interaction of the user with the application. Table 2 briefly describes each action associated to the arrows. Note that the the sequence of transactions depicted in Fig. 2 matches the toy example’s steps described in the supplementary material. The data flow diagram also shows the modular architecture of the software that permits developers to easily extend the code adding another type of functionality.

**Table 2:** List of actions associated to data flow diag ’s numbered arrows

Arrow number	Action
1	User’s interaction with the save/load menu
2	User’s interaction with the data to be loaded
3	Data visualization: handling
4	Data visualization: processing
5	Tracking procedure: handling
6	Tracking procedure: saving
7	Performance evaluation
8	User’s interaction with the data do be loaded as ground truth
9	Performance evaluation: file generation
10	User’s interaction with the data rendering/GUI’s appearance

### Volume visualization

Tools for volume visualization permit to set the ROI and offer some basic image processing functionalities, which are fully detailed in sections IV and V of the supplementary material.^3^ By default, the negative of the original grey level image is shown to enhance white or grey details embedded in dark regions, as in microscopic images they usually dominate in size.

When the 4*D* data is pretty huge, visualization and interaction can decrease the user experience. Hence, there is the chance to select a 3*D* ROI of the volume by setting in the upper right panel the voxel intervals for the *x*–*y*–*z* axes or by using the mouse directly in the GUI. Note also the such a ROI also helps user focus the attention to a certain volume.

On the visualized volume, Visual4DTracker permits to apply three image processing operations to improve the perceived rendering quality. The first allows to set to zero the opacity of all the voxels whose visualized grey level intensity exceeds a threshold, set by the user. This functionality removes some artifacts in the image and filters out possible salt and pepper noise. The second operation applies a transparency to all the voxels in the visualized volume regardless of their grey level intensity, setting $\tau$ in $[-1;+ \infty )$. If $\tau$ lies in $[-1;0]$, the software sets the opacity of all the voxels to $1 + \tau$. If $\tau >0$, the opacity of each voxel at coordinate (*x*, *y*, *z*) is set to $i^{\tau }(x,y,z)$, where *i*(*x*, *y*, *z*) is the original grey level voxel intensity. Hence, the brighter the voxel, the larger the transparency. This helps the user visualize darker objects in the image, which usually correspond to particles. This operation also removes the noise usually present in the smooth borders of the blobs, facilitating the detection of a single blob in a cluster.

The third operation linearly interpolates the intensity of the voxels, improving the spatial resolution and, in some cases, the perceived smoothness of the image. Note that previous 3*D* ROI selection reduces the computational burden of the interpolation.

### Handling, proofreading and evaluating the traces

**Fig. 3:**
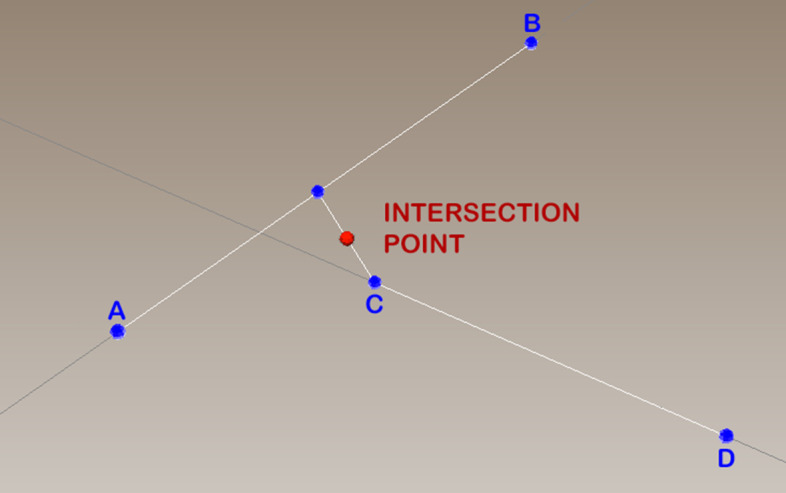
Graphical representation of the blob identification procedure in the software: the first user’s click on the blob in the 3*D* space will produce the piercing segment $\overline{AB}$; once the volume is rotated, the user performs a second click on the same blob which yields the piercing segment $\overline{CD}$ and the blob marker is set to the middle point of the minimum distance segment linking the two piercing segments

Visual4DTracker inherently works with $3D+t$ images. For a given *t*, the user can mark each blob position directly in 3*D* via two left mouse clicks as the intersection in $\mathfrak {R}^{3}$ between two incidents segments, as depicted in Fig. 3. To this aim, the first user’s click on the blob will produce a first piercing segment whose direction lies on the line orthogonal to the visualization plane (segment $\overline{AB}$ in the figure). Once the volume is rotated, the user performs a second click on the same blob which yields a second piercing segment (segment $\overline{CD}$ in the figure). However, since this procedure usually provides two skew segments, the blob marker is set to the middle point of the minimum distance segment linking the two piercing segments (the red dot in the figure). If needed, a right click on a detected particle deletes the corresponding marker.

To mark the positions of a blob over time the user can repeat the aforementioned two-left-clicks procedure for all the different *t* in the 4*D* stacks. It is also possible to show more than one trace in the same window.

As a key feature the software helps user proofread traces loaded from an external file, whose content is formatted as reported in the supplementary material. This can be very helpful as in many applications some existing software (e.g. TrackMate in Fiji) can be employed to trace the blobs: however, although in some cases they offer the possibility to tune the algorithmic parameters, the results can be affected by errors. In these cases, proofreading is a fast approach to correct the errors and move forwards with data analysis. To this aim, the aforementioned two-clicks procedure permits to modify blob markers positions. Visual4DTracker would help user do this job via the offered volume visualization functionalities in conjunction with the chance to select the traces and the corresponding blobs under investigation. In sections VI and VII of the supplementary material the interested readers find a detailed description of these operations and of the corresponding use cases.

The possibility to evaluate the tracing performance is another useful functionality. Each trace available in the workspace can be compared against a gold standard, loaded from an external file. For a proper comparison, we need to ensure that each predicted blob is uniquely associated with one gold standard blob, respecting also the temporal evolution of the movements. For this purpose, let us denote with *C* and *G* the set of marker positions for a trace in the test set and for the corresponding trace in the gold standard. The software first constructs an undirected bipartite graph with vertex set $C\cup G$. For each pair ${ \vec{c} }\in C$ and ${\vec{g}} \in G$ it adds an edge with weight

$$w_{{cg}} = \frac{1}{{\epsilon + {||}\vec{c} - \vec{g}{||}}}{\text{,}}\quad {\text{if}}\,||\vec{c} - \vec{g}|| < d$$

being *d* a distance parameter that can be roughly estimated as the expected diameter of an object, and $\epsilon$ a small constant preventing numerical overflows. It then computes the maximum weight bipartite matching. A test set marker position $\vec{c}$ is considered to be a *true positive* (TP) if it is matched to a gold standard position $\vec{g}$ such that $\Vert \vec{c} -\vec{g}\Vert < d/2$. Unmatched predictions are counted as *false positives* (FP) and unmatched gold standard markers are counted as *false negatives* (FN). From these metrics, we derive the precision (*P*), recall (*R*), and $F_1$ score as

$$\begin{aligned} P&=TP/(TP+FP) \\ \\ R&=TP/(TP+FN) \\ \\ F_1&=\frac{2P\cdot R}{P+R} \end{aligned}$$

Section IX of the supplementary material describes in practice how to evaluate the performance.

## Results

In this section we show how to use the Visual4DTracker toolbox and we report the results of its application to three applicative examples.

The first case is fully presented in the supplementary instruction manual, and it is a step-by-step toy example that guides the user through all the main phases. It is the synthetic construction of the random walk of 5 equally sized spherical blobs into a 4D space with dimensions of $50 \times 50 \times 50 \times 100$. Moreover, in order to better simulate the acquisition process, salt & pepper noise has been added to the entire stack. This example describes how to load an untraced 4*D* stack of synthetic blobs, how to use parameters’ tuning, blobs’ tracking, traces’ file saving, how to proofread the tracks’ and, finally, how to evaluate the tracking performance. For the sake of clarity all the steps are also shown in four self-explaining videos, which can be downloaded from the same web repository where the reader can find the instruction manual.

The second applicative example is offered as a video still available in the aforementioned web repository. It is based on a real 4*D* image stack showing the time-laps growth of Drosophila cells in an embryo. A representative screenshot of such task is shown in Fig. 1, which also reports the link to download the original repository. The 4D stack dimensions are $275 \times 300 \times 50 \times 50$. It is worth noting that the Drosophila images shown in the videos have a high-contrast and low noise, two desirable properties that simplifies image annotation attained by fine tuning the parameters of the proposed tool. Indeed, the original images are affected from a background white noise often cluttering the scene.

After presenting how the user can visualize and manage the image volume in the GUI, the video focuses on how to trace and to proofread a given cell trace.

The third example deals with the use of this software toolbox in diabetes research and it is fully presented hereinafter.

These three experiments permit us also to test the concrete usability and the responsiveness of the tool. Using a 3.1 GHz Intel i7 MacBook Pro equipped with 16GB RAM and a NVME SSD, Visual4DTracker opens and interactively shows without lag on a $50 \times 50 \times 9 \times 150$ ROI, selected using the tool from a $348 \times 260 \times 9 \times 150$ with more than 100 tracks. Furthermore, the interested reader can enjoy the effective and realistic no laggy experience offered by the tool watching the self explanatory videos in the web repository.

### 4D quantitative analysis of insulin granules in living beta-cells

The role of beta cells in the pancreas is to biosynthesize insulin and store it within inside granules (IGs) which are special vesicles for its packaging and glucose-stimulated secretion. In order to avoid Type 2 Diabetes Mellitus and other complications of the metabolic disease [12, 13], these delicate functions are highly regulated, and their defects can therefore cause impaired insulin secretion. The investigation of IG dynamics has therefore attracted research efforts in the last decades: initially, insulin secretion was measured biochemically in whole animals and individuals, but these studies did not furnish insight into the insulin granule subcellular trafficking following stimulation. To elucidate these mechanisms, several imaging techniques were developed and employed as powerful tools for detecting granule diffusion and trafficking [14, 15]. However, only the raise of spinning disk confocal and light sheet microscopy with fast sequential scanning [16, 17] has enabled rapid volumetric imaging, allowing high resolution IG tracking and a detailed reconstruction of the intracellular trajectories that contain more information than the mean values extracted from the aforementioned traditional approaches. Nevertheless, an approach for 4*D* IG tracking and analysis is still at infancy, being often limited either to local processing or to 2*D* image analysis. Indeed, the extraction of quantitative information relies upon the possibility to track the particles within a single cell with a satisfactory accuracy.

In this respect, Visual4DTracker has been used to carry out the quantitative analysis of the 4*D* motion of insulin granules in glucose-stimulated INS-1E beta cells. Details on the dataset, cell cultures, glucose stimulation, and image acquisition are reported in [18]. To detect and track IGs we developed a computer-based automatic approach based on a two-step iterative process that uses TrackMate, a freeware package available within the Fiji software platform [19]. The first step detects the (*x*, *y*, *z*, *t*) position of all the granules, whilst the second step computes a cross-frame connection, where the point $P_f$ identifying the granule *g* at frame *f* is linked with the point $P_{f+1}$ at frame $f + 1$ when $P_{f + 1}$ is considered the most likely identification of *g* on the frame $f+1$.

**Fig. 4:**
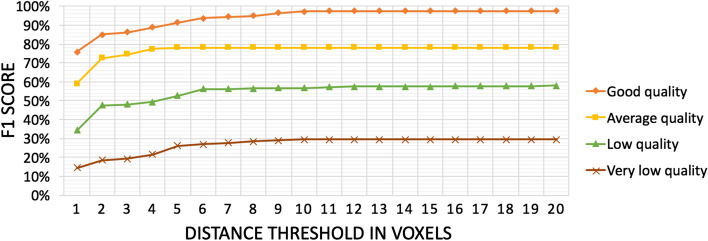
Automatic tracking quality evaluation

All 4D acquisitions showed low contrast being affected by noise despite the efforts to set the best combination of microscope’s parameters; as a consequence the images have poor quality, impacting also the granule detection stage that, in turn, could degrade the further quantitative analysis. We are therefore interested in finding out the performance of the tracking algorithm and, to this goal, Visual4DTracker played a fundamental role. Indeed, the toolbox was used to manually proofread a set of 100 traces selected among four different groups defined according to the duration of the traces, which can be considered a raw “quality” criterion of the results provided by the tracking algorithm. The four groups are: (1) a *very low quality subset* containing all the tracks satisfying $L \le 0.25F$, where *L* is the length in frames of the trace and *F* is the average number of frames per trace in the dataset, (2) a *low quality subset* composed of all the traces for which $0.25F < L \le 0.50F$, (3) an *average quality subset* containing all the traces for which $0.50F < L \le 0.75F$ and (iv) a *good quality subset* composed of all the traces satisfying $L \ge 0.75F$. For each group, 25 traces were randomly selected and manually proofread by two domain experts, who annotated the *x*, *y*, *z* and *t* positions for each of the selected IGs, obtaining the gold standard used to measure the tracing performance according to the Visual4DTracker procedure described in previous section. The average results measured in terms of F$_{1}$ score per each subgroup are shown in Fig. 4, suggesting us to do not consider the traces belonging to the *very low quality* and the *low quality* subsets since the detected positions are not reliable enough to represent the IG dynamics. We deem that our annotation overcomes most the existing limitations introduced by the use of any available open-source software that, to the best of our knowledge, permits to manual track the data performing only a frame by frame segmentation within a single layer of the stack. This approach allows only users tracking the granules in 2D, a task limited by its very nature, by the acquisition noise and by the overlap of multiple granules that prevent to correctly determine the correct particle direction.

**Fig. 5:**
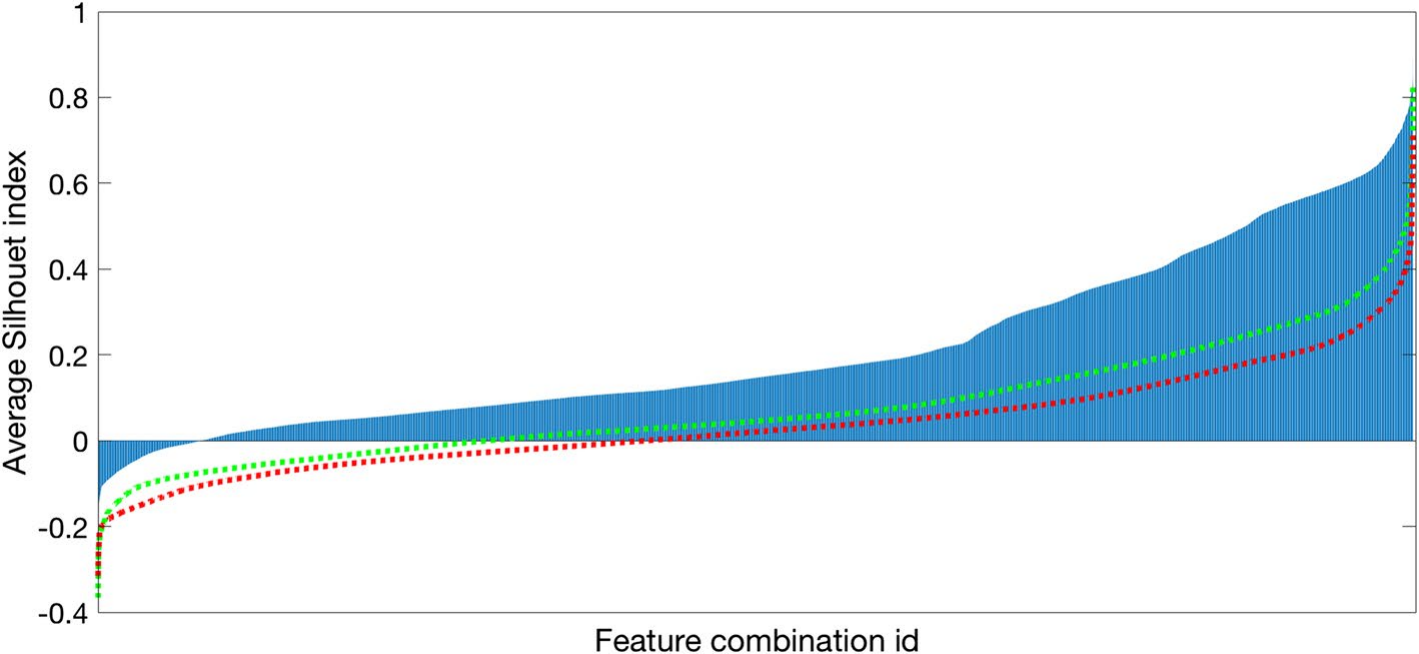
Overlap of sorted *Silhouette indexes* averages vs. the number *k* of clusters identified from the clustering algorithm. In blue is depicted the bar plot calculated for $k = 2$, in green and red are shown the trends obtained with $k = 3$ and 4 respectively

**Fig. 6:**
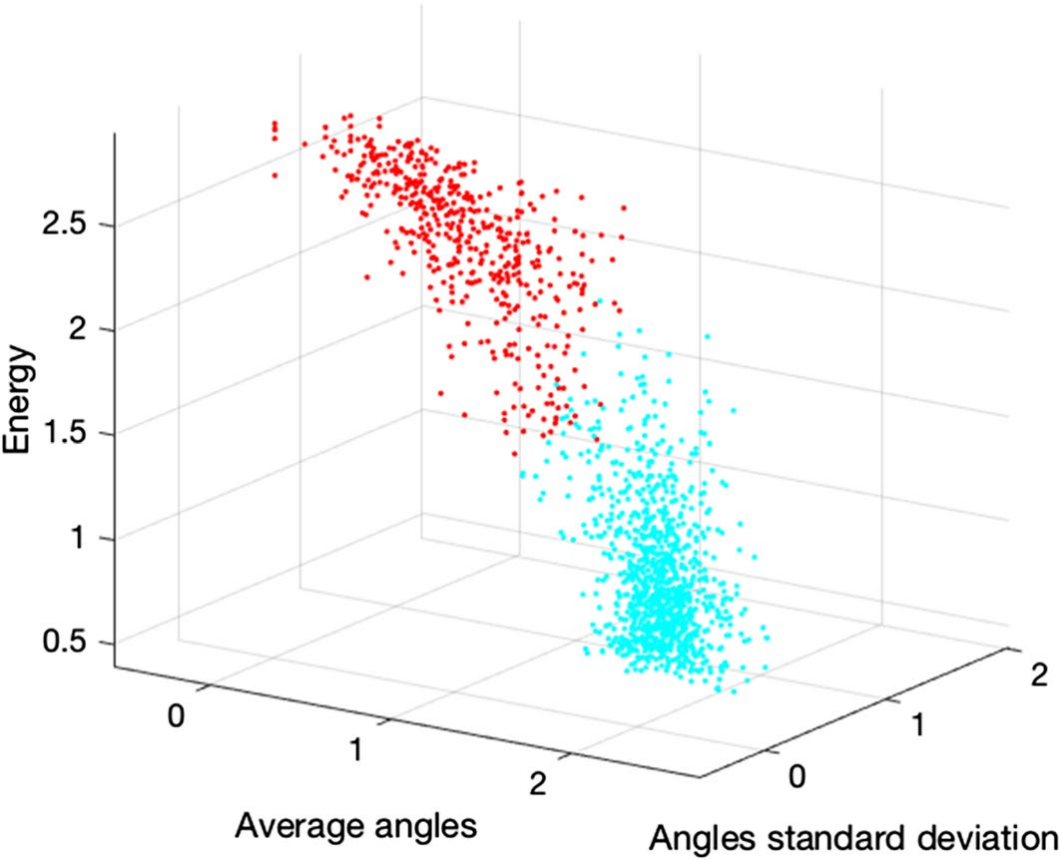
Scatter plot of the tracks after the clustering analysis and the feature selection. The two clouds of points are depicted in different colors

On the basis of such results attained thanks the use of toolbox presented here, we quantitatively described the IG dynamic behavior of all the traces belonging to the average and good quality subsets by computing 14 features. Such descriptors belong to two groups: the first, named as *route features* describes the route of the granule, whereas the second, named as *time dependent features*, computes quantities related to the hourly law of the motion. For the sake of completeness, Table 3 summarizes the formal definition of such features. Finally, these features are used to search for the number of clusters in the data: Fig. 5 reports the values of the *Silhouette index*, i.e. a popular clustering performance metrics [20], computed for all $2^{N}-1$ features combination (with *N* being the number of features) and for three possible number of clusters (2, 3 and 4). Such results suggest that the data contain two clusters. Furthermore, we found that *Average angles*, *Angles standard deviation* and *Energy* are the features that best cluster the data. They were detected ranking all the performance computed by the Silhouette index and by the *Davies-Bouldin index* [21], the *Calinski-Harabasz index* [22] and the *Simmetry Distance between and within index* [23] over all possible combinations. Figure 6 shows the scatter plot, where the two colors correspond to the two clusters found by the *Expectation Maximization* algorithm, which was selected after a preliminary investigation.

**Table 3:** Formal definition of computed features

Preliminary symbols definitions	
$\mathbf{F}$ is the set including the index of frames which contain one marker of the granule under study	
$\mathbf{x}$, $\mathbf{y}$ and $\mathbf{z}$ are vectors containing the coordinates of the marker of the granule under study at each frame $f \in \mathbf{F}$	
${\mathbf{p}} _f = ({\mathbf{x}} _{f}, {\mathbf{y}} _{f}, {\mathbf{z}} _{f})$, where $f \in [0;|{\mathbf{F}} |-1]$	
Set of displacements: ${\mathbf{D}} = \{ ||{\mathbf{p}} _{f + 1} - {\mathbf{p}} _{f}||, f \in [0;|{\mathbf{F}} | - 1]\}$	
$pdf(\cdot )$: probability density function of $\cdot$	
Set of angles: ${\mathbf{R}} = \left\{ \arccos \left( ({\mathbf{p}} _{f + 1} - {\mathbf{p}} _{f})\cdot ({\mathbf{p}} _{f + 2} - {\mathbf{p}} _{f + 1})\over {||{\mathbf{p}} _{f + 1} - {\mathbf{p}} _{f}||\cdot ||{\mathbf{p}} _{f + 2} - {\mathbf{p}} _{f + 1}||} \right) , f \in [0;|{\mathbf{F}} | - 2] \right\}$	
Set of velocity: ${\mathbf{V}} = \left\{ {\frac{{{\mathbf{P}}_{f} }}{{{\mathbf{F}}(f + {\mathbf{1}}) - {\mathbf{F}}(f)}},f \in [0;|{\mathbf{F}}| - 1]} \right\}$	
Set of acceleration: ${\mathbf{A}} = \left\{ {\frac{{{\mathbf{V}}_{{{\mathbf{F}}_{f} }} }}{{{\mathbf{F}}(f + {\mathbf{1}}) - {\mathbf{F}}(f)}},f \in [0;|{\mathbf{F}}| - 2]} \right\}$	

Features	
*Route-based*	*Hourly law-based*
Total displacement $(\Delta ) = \sum _{i = 1}^{|{\mathbf{D}} |} {\mathbf{D}} _i$	Average velocity $\left( \bar{{\mathbf{V }}} \right) = {{1}\over {|{\mathbf{V}} |}} \sum _{i = 1}^{|{\mathbf{V}} |} {\mathbf{V}} _i$
Average angles $\left( \bar{{\mathbf{R }}} \right) = {{1}\over {|{\mathbf{R}} |}} \sum _{i = 1}^{|{\mathbf{R}} |} {\mathbf{R}} _i$	Velocity standard deviation $= \sqrt{{{\sum _{i = 1}^{|{\mathbf{V}} |} ({\mathbf{V}} _i - \bar{{\mathbf{V} }})^2}\over {|{\mathbf{V}} |} - 1}}$
Angles standard deviation $= \sqrt{{{\sum _{i = 1}^{|{\mathbf{R}} |} ({\mathbf{R}} _i - \bar{{\mathbf{R} }})^2} \over {|{\mathbf{R}} |} - 1}}$	Velocity skewness $= {{{{1}\over {|{\mathbf{V}} |}}\sum _{i = 1}^{|{\mathbf{V}} |}({\mathbf{V}} _i - \bar{{\mathbf{V }}})^3}\over {\left( \sqrt{{{1}\over {|{\mathbf{V}} |}} \sum _{v = 1}^{|{\mathbf{V}} |}({\mathbf{V}} _i - \bar{{\mathbf{V} }})^2}\right) ^3}}$
Tortuosity $= {{\Delta }\over {|{\mathbf{p}} _{|{\mathbf{F}} |-1} - {\mathbf{p}} _1|}}$	Velocity kurtosis $= {{{{1}\over {|{\mathbf{V}} |}}\sum _{i = 1}^{|{\mathbf{V}} |}({\mathbf{V}} _i - \bar{{\mathbf{V} }})^4}\over {\left( {{1}\over {|{\mathbf{V}} |}}\sum _{i = 1}^{|{\mathbf{V}} |}({\mathbf{V}} _i - \bar{{\mathbf{V} }})^2\right) ^2}}$
Energy $= pdf({\mathbf{x}} )^2 + pdf({\mathbf{y}} )^2 + pdf({\mathbf{z}} )^2$	Average acceleration $\left( \bar{{\mathbf{A} }} \right) = {{1}\over {|{\mathbf{A}} |}} \sum _{i = 1}^{|{\mathbf{A}} |} {\mathbf{A}} _i$
Entropy $= -(pdf({\mathbf{x}} )\log _2{(pdf({\mathbf{x}} ))}~+$	Acceleration standard deviation $= \sqrt{{{\sum _{i = 1}^{|{\mathbf{A}} |} ({\mathbf{A}} _i - \bar{{\mathbf{A} }})^2}\over {|{\mathbf{A}} |} - 1}}$
$+~pdf({\mathbf{y}} )\log _2{(pdf({\mathbf{y}} ))}~+$	Acceleration skewness $= {{{{1}\over {|{\mathbf{A}} |}}\sum _{i = 1}^{|{\mathbf{A}} |}({\mathbf{A}} _i - \bar{{\mathbf{A} }})^3}\over {\left( \sqrt{{{1}\over {|{\mathbf{A}} |}}\sum _{i = 1}^{|{\mathbf{A}} |}({\mathbf{A}} _i - \bar{{\mathbf{A} }})^2}\right) ^3}}$
$+~pdf({\mathbf{z}} )\log _2{(pdf({\mathbf{z}} ))})$	Acceleration kurtosis $= {{{{1}\over {|{\mathbf{A}} |}}\sum _{i = 1}^{|{\mathbf{A}} |}({\mathbf{A}} _i - \bar{{\mathbf{A} }})^4}\over {\left( {{1}\over {|{\mathbf{A}} |}}\sum _{i = 1}^{|{\mathbf{A}} |}({\mathbf{A}} _i - \bar{{\mathbf{A} }})^2\right) ^2}}$

From a biological perspective, these results show the presence of two distinct families of granules coexisting inside the beta-cells that are identifiable by their different dynamics. The first is characterized by a diffusive motion, showing a larger angular average and a lower standard deviation of their acceleration at the same time, but resulting to have a greater movement energy (in cyan in Fig. 6). The second group includes granules with a more linear dynamics, which exhibits a lower angular average, as well as lower entropy and a more spread acceleration distribution, suggesting that they are characterized by a tubular motion.

## Conclusions

The Visual4DTracker is unique in the category of the tools for 4*D* image interaction because, even for non expert in image processing, it facilitates the analysis and annotation of such images by presenting a user-friendly 3*D* data representation. This can help analyzing time-lapse microscopy images that permit researchers to study the biological phenomena characterized by a 4*D* behavior. The use of the toolbox has been discussed in three applicative examples, which range from synthetic demo to a real application in diabetes research where Visual4DTracker played a key role to quantitatively analyse insulin granules dynamic in living beta-cells. Finally, this novel toolbox can be used also in any other domain where there is the need to visualize, analyse and annotate 4*D* images containing objects.

Future work can be directed towards porting the tool to Java to be incorporated into Fiji as a plugin.

## Availability data and requirements

- Project name: *Visual4DTracker*
- Project home page: https://drive.google.com/drive/folders/19AEn0TqP-2B8Z10kOavEAopTUxsKUV73?usp=sharing
- Operating system(s): Windows, Unix and Linux
- Programming language: MATLAB
- Other requirements: MATLAB 2015 or latest versions
- License: MATLAB

## Supplementary information

**Additional file 1.** Visual4DTracker Instruction Manual. In order to practically familiarize with the aforementioned utilities, the interested reader can find more detailed instructions in the supplementary material, which will guide the user through the software with a step by step load, proofread and performance evaluation of a freely downloadable toy image stack.

## Data Availability

The most recent version of the software and of its documentation is publicly available here: https://drive.google.com/drive/folders/19AEn0TqP-2B8Z10kOavEAopTUxsKUV73?usp=sharing.

## Abbreviations

FN: False negative(s)
FP: False positive(s)
GUI: Graphical user interface
IG: Insulin granule
MW: Main window
P: Precision
R: Recall
TP: True positive(s)

## Footnotes

1 A schematic example of the *xml* format model is presented in the Appendix of the “Visual4DTracker Instruction Manual”.

2 For a detailed explanation of how to generate the correct format output files we refer the reader to DiaTrack’s [8] and ilastik’s [9] guidelines.

3 As detailed in the supplementary material, the user can add to Visual4DTracer any image processing filter coded in an MATLAB .m file.
