# Supplementary material for: Visual4DTracker: a tool to interact with 3D + t image stacks
Source: BMC Bioinformatics. 2021 Feb 8;22:53. doi: 10.1186/s12859-020-03820-y (PMC7869512; doi:10.1186/s12859-020-03820-y)
Supplement: Supplementary file 1 — Additional file 1. Visual4DTracker Instruction Manual. In order to practically familiarize with the aforementioned utilities, the interested reader can find more detailed instructions in the supplementary material, which will guide the user through the software with a step by step load, proofread and performance evaluation of a freely downloadable toy image stack. [file 12859_2020_3820_MOESM1_ESM.pdf]

Visual4DTracker Instruction Manual

Ermanno Cordelli, Paolo Soda and Giulio Iannello
Unit of Computer Systems and Bioinformatics, Department of Engineering,
Università Campus Bio-Medico di Roma, Rome, Italy
{e.cordelli, p.soda, g.iannello}@unicampus.it

I. INTRODUCTION

This *step-by-step* supplementary note shows how to use Visual4DTracker, a software that allows to handle, to visualize and to surf 4D stacks of images, containing objects[1] moving in a 3D space and acquired over time at a microscope. Moreover, the Graphical User Interface (GUI) of the tool allows the user to mark the objects' positions frame by frame and to save their traces in a file that can be exported; there is also the possibility to load a file containing a set of traces and to proceed further with data annotation. This functionality yields the possibility to proofread traces already marked by other users or provided by a software for automatic tracing, thus being able to compare two different traces belonging to the same experiment.

This quick guide intends to support the reader trough the use of the software, and it therefore makes use of a toy (synthetic) example to present how the user can interact with the GUI, load a stack, handle the 4D volume, visualize the data, handle and define traces, and assess the tracing performances. The presentation of the toy example is offered in the light blue boxes in the following. Furthermore with the intent of describing the processes given by the interaction of the user with the GUI, this supplementary material in the light red boxes refers to figure 2 of the main manuscript, which depicts the data flow diagram. The software as well as the data of the toy example can be downloaded here. Furthermore, some videos presenting this tool are available at the same link.

II. INSTALLATION

The software tool is a Matlab package written using the R2015b version of the software. It is therefore a cross-platform application working on Windows, Unix and Linux operating systems and, aside from Matlab, it does not require any further installation.

Visual4DTracker accepts a series of stacks of 2D grayscale images[2] of any Matlab supported format, i.e. *BMP*, *GIF*, *HDF*, *JPEG*, *JPEG2000*, *PBM*, *PCX*, *PGM*, *PNG*, *PNM*, *PPM*, *RAS*, *TIFF* and *XWD*.

III. LOADING OF 4D STACK AND TRACES

To run the application navigate to the "Visual4DTracker" folder and type "Visual4DTracker" in the Matlab Command Window. Once the software has been launched, the main GUI with all the panels disabled will appear, as shown in Fig. 1.

The user can interact with the global features of the application using the items located in the menu list on the top of the GUI, marked by (1) in Fig. 1.

First, to load a 4D stack click on the "File" menu and then on the "Load" > "Stack.." item. A dialog box pointing to the current Matlab's folder will appear, but the user has to select the main folder containing the data of the experiment. Note that the application will read and write data (being either the 4D volume, the traces, the report on the assessed performances or the log files) located only in that main folder. Such a folder is conceptually a frames' container, that are actually stacks of 2D images stored in subfolders and where each image is a slice of the same stack. Each subfolder corresponds to a given time step, and it has to be named as *name you'd like-time*. Furthermore, all the images must have the same sizes, and the number of images per subfolder cannot vary. Once the user has selected the main data folder containing all the stacks, click on the "Open" button in the dialog box. The application will load in memory the data contained in all the subfolders. Next, there is the possibility to specify the voxel spacing of the images and, by default, all the values are set to 1. There is also a chance to load an xlm file containing a set of traces already available for this stack at hand.

The described steps correspond to **arrow number 1** in the data flow diagram (figure 2 of the main manuscript).

This file could have been saved as presented in section VIII, and its format is described in the appendix.

To load the stacks of the toy example, click on "File" > "Load" > "Stack..", navigate to the "toy example" main folder, select the "4D images" subfolder and then click on the "Open" button.

[1] We use the general term objects, although in the literature they are often referred to as corpuscles or blobs.
[2] The maximum stacks' dimensions admitted are determined from the user's Matlab memory usage setting and depends on the color depth of images loaded.

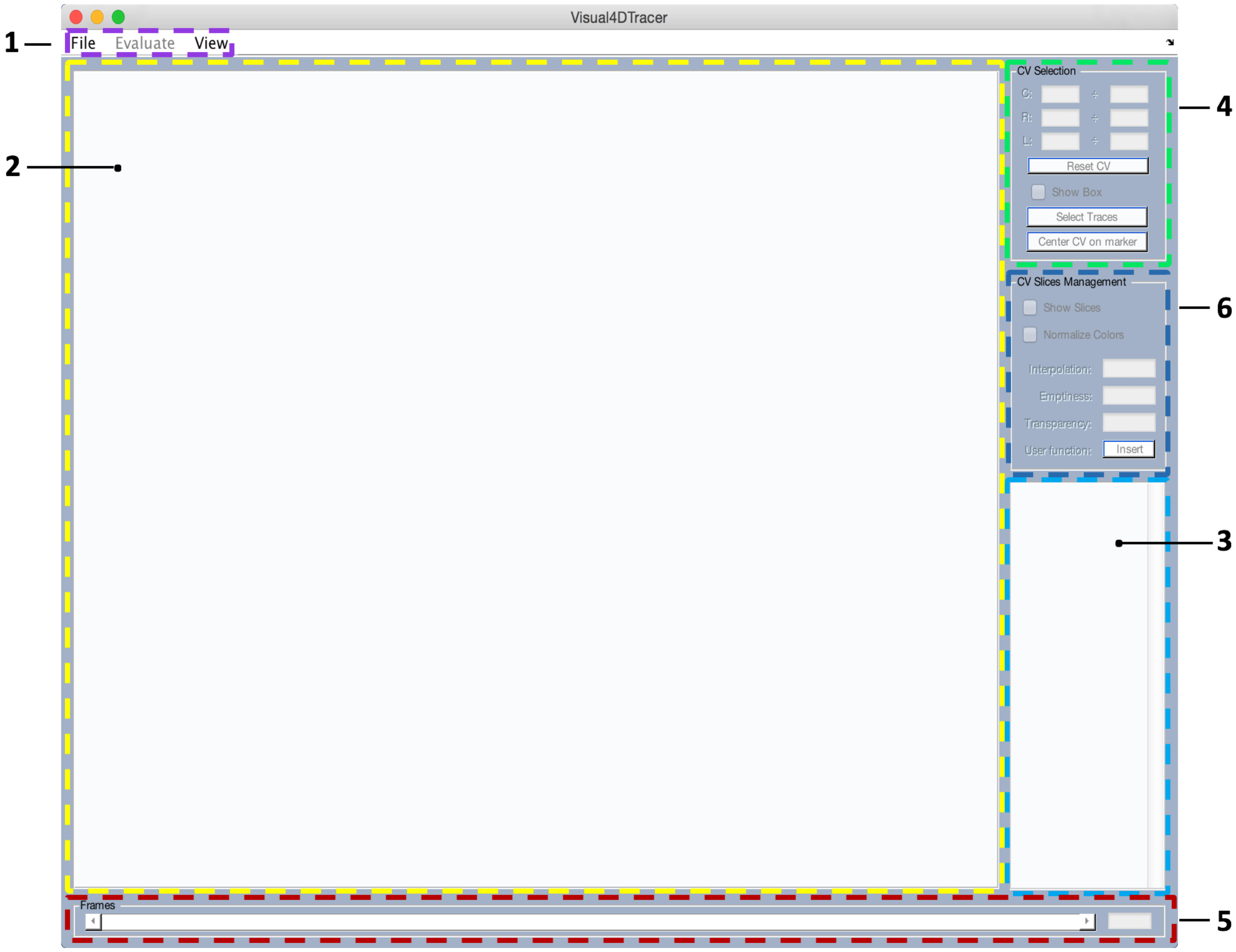

Fig. 1: Main GUI once the application has been launched. Different areas of the GUI are highlighted by different colors to facilitate its description trough the text.

To load the stacks of the toy example, click on "File" > "Load" > "Stack..", navigate to the "toy example" main folder, select the "4D images" subfolder and then click on the "Open" button. Next, keep the default values (1) for the voxel spacing and click "Ok". Now, to load the related traces, select the "traces.xml" file located in the same folder.

Once the dialog box has closed, the data are loaded in the GUI's variables workspace, and within the GUI main panel (2) by default the application shows only the empty euclidean space and the axes with scales, whose ranges fit with the stack dimensions.

The described steps correspond to **arrow number 2** in the data flow diagram.

This allows to unburden the graphics memory, while waiting for the user who can set up the desired point of view and the portion of the volume to be shown by selecting a control volume (CV) within the whole stack. This procedure will be described in section IV. Furthermore, the slide bar at the bottom of the GUI is set to the first frame.

The user should now interact with the trace panel, numbered as (3) in the figure. This panel lists all the traces reported in the xml file; note that the last element of the list, named as "new trace" corresponds to an empty trace and it can be used to add a new trace, as described in section VII.

The toy example contains 4 traces, and therefore the fifth item in the list allows to add a new trace.

IV. VOLUME HANDLING

Once the data are loaded, GUI panels will be enabled and they allow to interact with the 4D stacks. To this aim, the user works with the *CV Selection* (panel (4) in Fig. 1), where he/she can set the boundaries of a cropped sub-volume of the data.

By default, the ranges for each of the three C, R and L axes[3] reported in the text-boxes are equal to the ranges shown in the main window. Furthermore, to visualize within the main window the selected sub-volume the user has to select the *show box* toggle button: the boundaries of the parallelepiped region are reported in red, green and blue, which correspond to R, C and L, respectively. To set the boundaries of the sub-volume to be shown, the user can set the CV principal diagonal coordinates by typing the values in six text boxes. For each of the three axes (denoted as C, R and L in the figure) the first text box corresponds to the sub-volume corner closest to the origin, whilst the second box corresponds to the farthest corner, as depicted in the example shown in Fig. 2. Alternatively, the user can set the boundaries using the main window: to this aim, right click on the *main panel* at a desired location, than a contextual panel will appear allowing the user to select which of CV's borders to shift at clicked coordinates.

If you have already selected a blob within a trace (as described in sections VI and VII) there is also the possibility to automatically center the parallelepiped region at the selected blob in the selected frame; furthermore the size of the parallelepiped is equal to the values shown in the C, R, L text boxes. To use this functionality press the "Center CV on marker" button or use the key *F*.

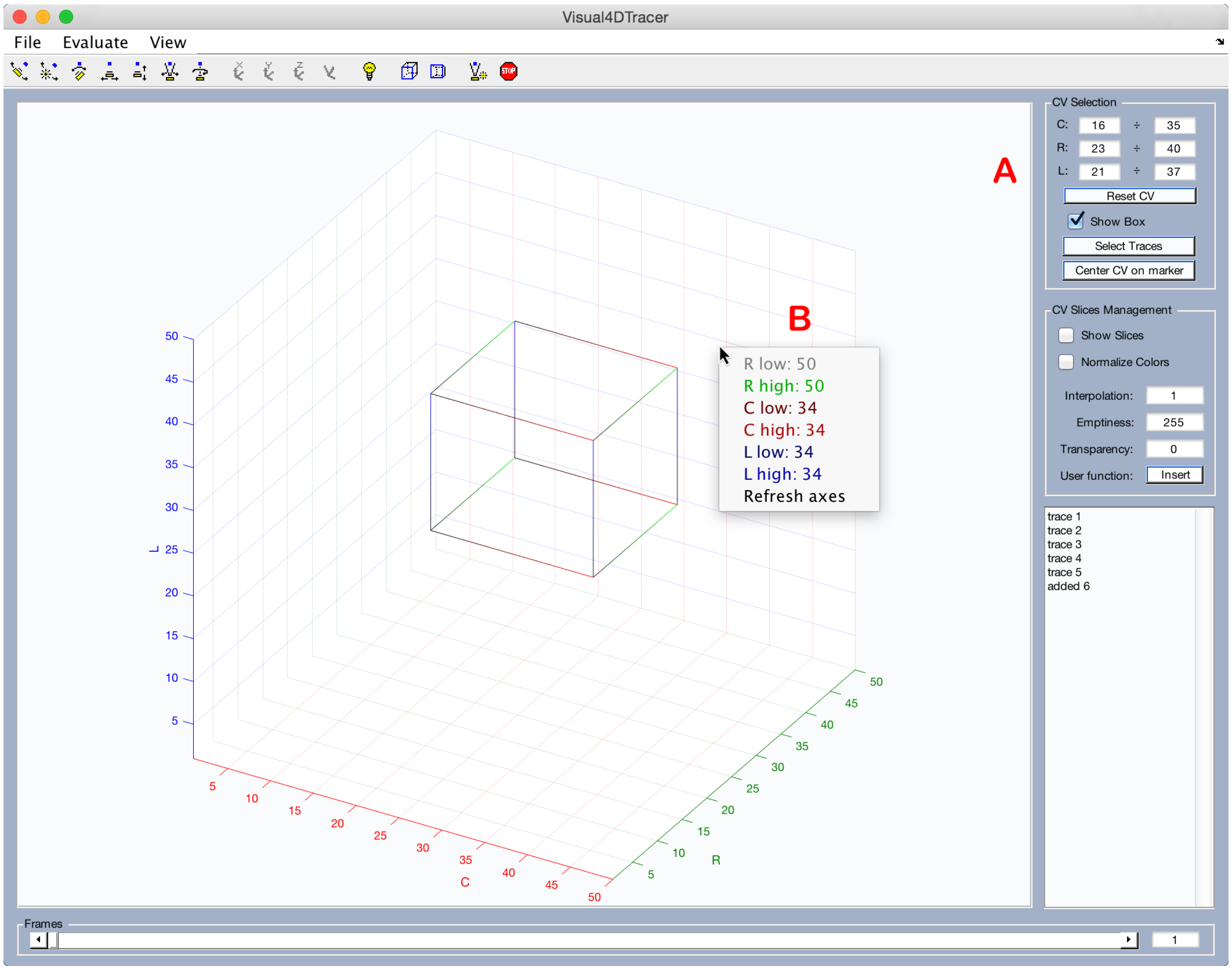

Fig. 2: CV boundaries definition in the "toy example": A) using *CV Slices Managment* panel or B) using *main panel*'s contextual menu.

The tool also allows users to explore the 4D stack in space and in time. Indeed, the user can *rotate* the volume around the point at the center of the main panel, *traslate* the stack and *zoom* in or out. These operations can be performed using the navigation toolbar shown on the top of the main panel. To improve the visual experience and to speed up the process, rather than using the toolbar the user can rotate, translate or zoom the volume via three main shortcuts based on the *T* or *Y* or *U* keys, respectively, that must be held down while clicking and dragging the mouse around. As reported above, the temporal domain can be controlled using the *Frames* panel (5) at the bottom of the main window: it is possible to move the slider to

[3]C, R and L stand for columns, rows and layers, respectively, and the same labels are shown in the main window close to each axis.

scan the experiment frames or by typing the specific number in the bottom-right editable space. Moreover the user can use the *spacebar* to rapidly move the frame by one step forward and *shift* + *spacebar* to move one step backward.

The described steps correspond to **arrow number 3** in the data flow diagram.

V. SLICE VISUALIZATION

Once the sub-volume of interest has been set, the user can visualize the portion of its slices within the main window by selecting the *Show Slices* toggle button in the *CV Slices Management* panel (6). The result is a box completely filled with all voxels belonging to the selected part of the volume, including the objects of interest of the experiment together with the background and, eventually, the acquisition noise. This panel is specifically designed to process the images and to remove from the stack all non informative elements as much as possible.

To enhance the quality of the image shown, the user can perform one (or at least all) of the three following procedures[4].

The first parameter is named as *Interpolation*, and it provides a sharper visualization of the whole sub-volume by performing a trilinear interpolation over all the voxels to simulate the increase or the reduction of the global level of detail of acquisition. The value in the edit box allows to set the distance of two consecutive planes normalized to the original distance, admitting values lying in [0.1; 10]. For instance, a value of 0.5 will insert an interpolated plane between each couple of original plane for all the dimensions of the stack, while a value equal to 3 will show one plane every two. Use this parameter wisely: a too thin interpolation will cause an high graphical computational load.

Try to set the interpolation value equal to 0.5 in the toy example.

Second, user can apply a threshold to voxels' intensity, removing all the voxels brighter than the threshold because part of the background. To this aim, use the *Emptiness* parameter, where the user can set a value within 0 and $2^b - 1$, where b denotes the number of bits used to represent the image intensity. Pixels whose intensity is larger than the value of the emptiness are set as transparent, and they are not visualized in the volume.

Try to set the emptiness value equal to 150 in the toy example.

Third, the user can set the *Transparency*, a less "aggressive" parameter that tunes the transparency of voxels whose intensity is below the emptiness. In the textbox, values must lie in $[-1; +\infty]$: when using a value in [-1; 0) the software reduces the global opacity of the visualized region. For instance, if you set -0.3, the opacity is reduced by 30%, i.e. the transparency is set equal to 70%.

When typing in the text box a value larger than 0, the voxels opacity is defined with a power law function of their normalized grey level intensity[5], where the user sets the power exponent. Its behavior is shown in Fig. 3.

Try to set the transparency value equal to 2 in the toy example.

The described steps correspond to **arrow number 4** in the data flow diagram.

VI. TRACES HANDLING

The panel in the lower-right corner permits to handle objects' traces (panel (3) in Fig. 1). It shows the list of all traces contained in the loaded file, that is four traces in the example. Let us recall that a trace records the center of an object moving in the space over time: hence, given a trace, it would be interesting to contemporary visualize the position of the object at a certain time and its trace from the beginning to the end of the observation.

As for the volume slices, once the data are loaded, all the traces are not shown in the axes: the user can plot in the main panel either the object's position marker or the line showing the whole object's positions marked inside all the frames, which in the following we refer to as route. The user can click the *G* key to show the object's position marker, after selecting on one or more traces from the list. To hide the marker, the user can simply use the combination *shift* + *G*.

Select the first trace from list and press *G*: a green circle located at the 3D coordinates of the trace will be shown in the corresponding frame, and it will be updated when frames changes.

To plot the entire route of the trace, the user first selects the trace in the list and then it presses the *R* key, whereas the combination *shift* + *R* should be used for hiding.

[4]In the following we assume to be working with images containing bright objects on a dark background.
[5]The grey level value of each voxel is normalized to the color depth of the stacks.

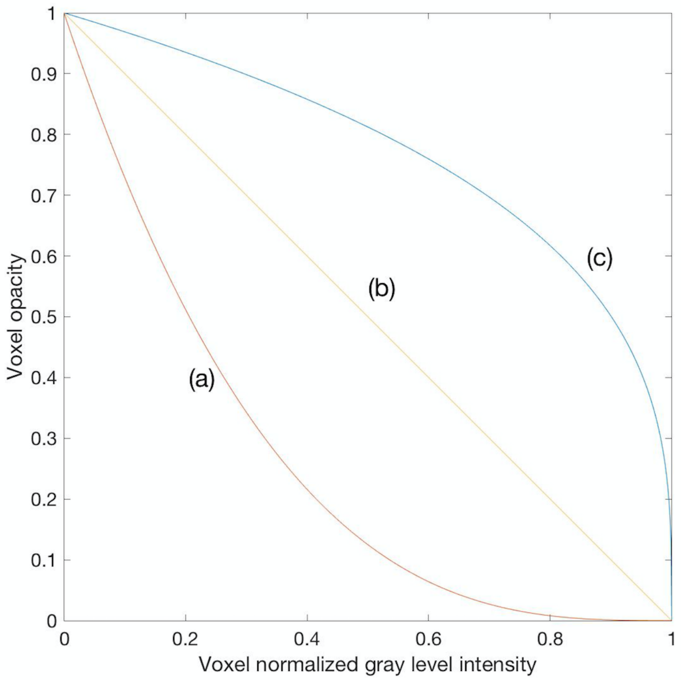

Fig. 3: *Transparency* non-negative behavior show. Voxel opacity vs. Voxel normalized grey level intensity using three different parameter's value: 3 (a), 1 (b) and 0.3 (c).

Press the *R* key on a trace: the software will show a black broken line, and the marker position will be updated when moving the frame slider.

If the user has selected a specific CV, traces outside the sub-volume are not of interest. It is possible to automatically select all the traced objects inside the box by clicking the *Select Traces* button at the bottom of the *CV Slices Managment* panel. This operation highlights in the trace list the traces contained in the CV, hence, the user can press *G* and *R* keys to visualize them in the main panel.

The last item in the list, "new trace", is reserved to the definition of a new trace, which is considered as a trace with no object spotted in any frame.

Finally, the user can add to "Visual4DTracker" any image processing filter: to this aim, press the *Insert* button and select the Matlab's .m file with the filter code. The algorithm of the added filter must process the entire stack frame by frame; furthermore, the input parameter of the function must be a 3D matrix represented in double format, and it must return the processed 3D matrix of doubles. To remove the filter press the same button, which is now named as *Remove*; furthermore, if the user puts the mouse over this button, the full path of the current filter will be shown in a while.

VII. TRACES DEFINITION

This package offers a set of functionalities to trace objects 4D movements in a 3D environment. The basic tool allows to generate points in the 3D environment, which can be used to add or to modify the object position at a given time stamp. To this aim, the user clicks twice the same point in the 3D space by two different points of view. The rationale of this technique lies in the fact that a point in the 3D space can be identified as the intersection of two coplanar non parallel lines. The first line is defined by the user mouse click on the target object: the software sets the line on the direction perpendicular to the screen plane at mouse coordinates. The user then changes the point of view by rotating the volume and defines the second line by clicking again on the target object (Fig. 4).

In practice, it seems unlikely the user sets two coplanar lines using the mouse, while it seems reasonable he/she sets two skew lines. On this ground, the target's coordinates are calculated as the middle point of the segment representing the minimum distance between the lines.

In the toy example set the frame number to 1 and focus your attention on the object closest to the ground, panning the object at the center of the screen to easily rotate it.

In the *traces list* now select the "new trace" item and press *G* to enable the visualization of markers you are defining. Hold down *T* and then hold down the mouse button to rotate the volume and to choose a desired point of view of the object, and

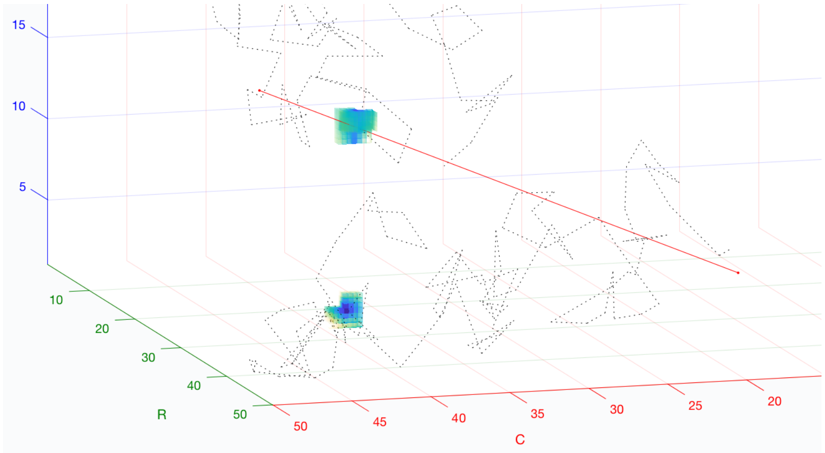

Fig. 4: Zoom over the object's marking procedure: the user has defined the first line (red) and rotated the camera to define the second line and mark the object in the stack.

then release *T*. Next use the combination *shift* (a cross will substitute the arrow pointer) + *mouse button* to set the first line: a red line perpendicular to the screen will appear. Now change the direction of the camera rotating the volume and define another line using again *shift* + *mouse button*: the old red line will disappear and a marker will be set at the line intersection point.

The package offers a fast way to do the same task. Once you have selected the "new trace" item and pressed *G*. You are in a first default view automatically set, and then use *shift* + *mouse button* to set the first line. Press *C* to move to a second default view and then define another line. Note that you can update the values of the angles' view by rotating the camera using *T* and the mouse button, as described before.

In the toy example set the frame number to 1 and focus your attention on the object closest to the ground, panning the object at the center of the screen to easily rotate it. In the *traces list* now select the "new trace" item and press *G* to enable the visualization of markers you are defining. Hold down *T* and then hold down mouse button to rotate the volume and choose a desired point of view of the object, and then release *T*. Next use the combination *shift* (a cross will substitute the arrow pointer) + *mouse button* to set the first line: a red line perpendicular to the screen will appear. Now change the direction of the camera rotating the volume and define another line: the old red line will disappear and a marker will be set at the line intersection point.

Now that the first marker is set, use the *Frames* panel to move to next frame (or press *space* once) where no markers exist and repeat the aforementioned procedure. Once you have marked the object's coordinates for a certain number of frames trying to follow the object's movement inside the stack, press the *R* key on the trace list after selecting the "new trace" item. The result will be a broken line connecting detected object positions, which represents object path in the space and defines the new trace.

The user can repeat all steps described above to define other new traces: to add an entirely new trace click the *add new empty trace* button at the bottom of the trace list.

Given a trace, no more than a marker per frame can exist: therefore, if you set a new marker in a frame where the marker already exists, the software overwrite the marker coordinates. This permits to proofread an existing trace, as well as to handle any possible annotation errors.

Finally, to delete a marker right click on any position in the the main volume area and select the item *delete marker* from the contextual menu. If you are interested in deleting the entire trace, first select a trace in the trace list, second, right click on it and, third, select the item *delete all markers* from the contextual menu.

At last, to distinguish an already analyzed trace from the others, in the trace list the user can right click on it and select the item "Mark as checked" from the contextual menu. This checking operation is mandatory to evaluate the performances, as described in IX.

The described steps correspond to **arrow number 5** in the data flow diagram.

VIII. SAVING 4D TRACES

Once the user has traced and/or proofread the object positions, the data can be saved in an xml file, allowing to write and to exchange text files containing all information on traces and markers 4D coordinates[6].

To this goal, click on the *File* menu and then on *Save* item: a dialog box pointing to the file folder where original data are stored will be shown. The user can choose a name for its file and save the traces.

When the number of objects to trace is quite large, or if the user needs to suspend the work, there is the possibility to save the traces, and then load it back later to continue. To agevolate this procedure, the user can export (and than import) the local variables of the GUI's setting.

To do it in our toy example click on "File" > "Export GUI's configuration file.." item, than choose a name and location for the file.

The file will contain all GUI's parameters set by the user before saving the local variables related to the *CV Slices Managment panel*, the *CV Slices Management panel*, the *Frames panel*, the traces list and the *View* menu.

To load back the local variables from a file, select "File" > "Import GUI's configuration file.." item and choose the previously exported file.

The described steps correspond to **arrow number 6** in the data flow diagram.

IX. PERFORMANCE EVALUATION

Given a stack, the software allows to estimate the accuracy of 4D object detection by comparing for all the traces the positions of their markers with the reference positions of the markers of the same stack. For simplicity, the reference set of traces is referred to as *gold standard*, whereas the set of traces under investigation is named as *test set*. In practice, the test set can be the output of any 4D tracing software, and the gold standard can be the proofread stack.

Select from the trace list the trace number 3, then press *R* and *G* to plot the route of the trace and the object's marker. Now navigate the frames 11 - 15 and notice that the markers do not correctly match the object's position in the stack. Please proofread the volume correcting the wrong marker positions in these five frames, and then save again the file (creating a new xml file or overwriting the existing one). Experiment also the possibility to check this trace as marked and to save the local variables in another file.

The described steps correspond to **arrow number 7** in the data flow diagram.

Now it is possible to evaluate the performance of the original trace number 3 with respect to the proofread one.

In the example, we can consider the original toy data as the test set and the new set of traces as the gold standard. First load the original toy data in the software, second click on the *Evaluate* menu item, third select the item *Load xml gold standard traces..*, fourth select the recently proofread and saved xml file, finally import the local variables' file associated to the traces: the software will compare only the checked traces in the list. Finally, select the *Evaluate performances..* menu item in the same *Evaluate* menu.

To properly compare test set positions against the gold standard we need to ensure that each test set marker is uniquely associated with at most one gold standard marker.

The described steps correspond to **arrow number 8** in the data flow diagram.

Given a trace and time t^*, let us denote with $(\mathbf{x}_m, t^*)$ and $(\mathbf{x}_g, t^*)$ the $4D$ coordinates for a predicted blob and a gold standard blob, respectively, where $\mathbf{x}$ stands for the $3D$ coordinates. A predicted blob is considered a *true positive* if $||\mathbf{x}_m - \mathbf{x}_g|| \leq d$, where d is a distance in pixels that the user can set exploiting a-priori knowledge on the biological domain under investigation. In the other case, the predicted blob is considered a *false positive*, whereas we have a *false negative* when the predicted blob is missing in comparison with the gold standard. As occlusions during blob movements can occur, the evaluation tool also counts how many times a blob disappears both in the predicted and in the gold standard traces. The results are saved in a csv file; at the beginning it reports a brief parameters description, followed by the the list of performance computed for each trace. Furthermore, it also reports the average value of all the performances scores.

[6]This format is adopted by many popular softwares, as *ImageJ*, *OsiriX*, etc.

In the previous example, once the gold standard has been selected, a window will pop up so that the user can set the value of D: for our toy data, set the value to 3 and press *Ok*. In the next dialog box, insert a name for the output file.

The described steps correspond to **arrow number 9** in the data flow diagram.

X. OTHER UTILITIES

The GUI allows to set other minor parameters related to how images and traces are rendered. With reference to Fig. 2, these options are located in the *View* menu and consist in the following five main items:

Axes: it allows to set the axes ratio. The default value is *Equal*.

Linetype: this changes the line-type of both markers and traces. The former can be represented as dot, circles (dimensionless markers) and balls (volumetric markers), whereas the latter are represented using solid or dashed lines. The default values are *circle* and *solid*, respectively.

Slices: it is composed by two items. The first, named as *Colormap*, changes the colormap and representation of slices. The implemented maps are *gray*, *gray inverted* (which inverts the gray color map), *highlighted* and *highlighted inverted* (which again inverts the colormap). The second item is named as *Direction* and it shows the slices along all the dimensions or only along the *Z axis* to alleviate the memory usage for big images. The default values are *gray* and *all*, respectively.

Render: it permits to select between two rendering engines, namely *OpenGL* and *Painter*. The former uses the hardware acceleration to perform faster image rendering; the latter produces a more detailed, but even slower, result. The default value is *OpenGL*.

GUI color: it permits to set basic theme colors for the GUI. The default value is *blue*.

The described steps correspond to **arrow number 10** in the data flow diagram.

APPENDIX

This appendix shows below a schematic example of the xml format model used in Visual4DTracker to read and store the traces coordinates in 4D.

Note that depending on objects' dynamic the occurrences of spots may not match with the duration of experiment, therefore FRAME_ID could not be stored as progressive consecutive indices. This can happen, for instance, when the delay between two consecutive frames is larger than one second.

Notations:

- NUM_TRACES: Number of traces contained in the file.
- DATE: File's creation or last modification date coded in "day, day_number month year hh:mm:ss".
- FRAME_ID, X_COORD, Y_COORD, Z_COORD frame id, X, Y and Z coordinates, respectively, written using a floating point type.
- NUM_SPOTS: Number of times each blob was detected.

```
<?xml version="1.0" encoding="UTF-8"?>
<Tracks nTracks="NUM_TRACES" spaceUnits="pixel" frameInterval="1.0" timeUnits="frame"
        generationDateTime="DATE" from="Visual4DTracker">
  <particle nSpots="NUM_SPOTS">
    <detection t="FRAME_ID" x="X_COORD" y="Y_COORD" z="Z_COORD" />
    <detection t="FRAME_ID" x="X_COORD" y="Y_COORD" z="Z_COORD" />
    <detection t="FRAME_ID" x="X_COORD" y="Y_COORD" z="Z_COORD" />
    <detection t="FRAME_ID" x="X_COORD" y="Y_COORD" z="Z_COORD" />
     ...
    <detection t="FRAME_ID" x="X_COORD" y="Y_COORD" z="Z_COORD" />
  </particle>
  <particle nSpots="NUM_SPOTS">
     ...
  </particle>
   ...
</Tracks>
```
